# Health Behaviors of Higher Education Students from 7 Countries: Poorer Sleep Quality during the COVID-19 Pandemic Predicts Higher Dietary Risk

**DOI:** 10.3390/clockssleep3010002

**Published:** 2021-01-15

**Authors:** Chen Du, Megan Chong Hueh Zan, Min Jung Cho, Jenifer I. Fenton, Pao Ying Hsiao, Richard Hsiao, Laura Keaver, Chang-Chi Lai, HeeSoon Lee, Mary-Jon Ludy, Wan Shen, Winnie Chee Siew Swee, Jyothi Thrivikraman, Kuo-Wei Tseng, Wei-Chin Tseng, Juman Almotwa, Clare E. Feldpausch, Sara Yi Ling Folk, Suzannah Gadd, Linyutong Wang, Wenyan Wang, Xinyi Zhang, Robin M. Tucker

**Affiliations:** 1 Department of Food Science and Human Nutrition, Michigan State University, East Lansing, MI 48824, USA; duchen@msu.edu (C.D.); imigjeni@msu.edu (J.I.F.); almotaw2@msu.edu (J.A.); feldp107@msu.edu (C.E.F.); folksara@msu.edu (S.Y.L.F.); gaddsuza@msu.edu (S.G.); wangliny@msu.edu (L.W.); wangwe60@msu.edu (W.W.); zhan1035@msu.edu (X.Z.); 2 Division of Nutrition and Dietetics, International Medical University, Kuala Lumpur 57000, Malaysia; megan_chong@imu.edu.my (M.C.H.Z.); winnie_chee@imu.edu.my (W.C.S.S.); 3 Global Public Health, Leiden University College, 2595 DG The Hague, The Netherlands; m.j.cho@luc.leidenuniv.nl (M.J.C.); j.k.thrivikraman@luc.leidenuniv.nl (J.T.); 4 Department of Food and Nutrition, Indiana University of Pennsylvania, Indiana, PA 15705, USA; pyhsiao@iup.edu; 5 Department of Kinesiology, Health, and Sport Science, Indiana University of Pennsylvania, Indiana, PA 15705, USA; hsiao@iup.edu; 6 Department of Health and Nutritional Science, Institute of Technology Sligo, F91 YW50 Sligo, Ireland; keaver.laura@itsligo.ie; 7 Department of Exercise and Health Sciences, University of Taipei, Taipei 11153, Taiwan; sports_injury0406@yahoo.com.tw (C.-C.L.); fossil0405@yahoo.com.tw (K.-W.T.); speedceng@gmail.com (W.-C.T.); 8 Department of Human Services, Bowling Green State University, Bowling Green, OH 43403, USA; leeh@bgsu.edu; 9 Department of Public & Allied Health, Bowling Green State University, Bowling Green, OH 43403, USA; mludy@bgsu.edu (M.-J.L.); wanshen@bgsu.edu (W.S.)

**Keywords:** sleep, dietary risk, physical activity, alcohol misuse, COVID-19, undergraduate and graduate students

## Abstract

Health behaviors of higher education students can be negatively influenced by stressful events. The global COVID-19 pandemic presents a unique opportunity to characterize and compare health behaviors across multiple countries and to examine how these behaviors are shaped by the pandemic experience. Undergraduate and graduate students enrolled in universities in China, Ireland, Malaysia, South Korea, Taiwan, the Netherlands and the United States (USA) were recruited into this cross-sectional study. Eligible students filled out an online survey comprised of validated tools for assessing sleep quality and duration, dietary risk, alcohol misuse and physical activity between late April and the end of May 2020. Health behaviors were fairly consistent across countries, and all countries reported poor sleep quality. However, during the survey period, the COVID-19 pandemic influenced the health behaviors of students in European countries and the USA more negatively than Asian countries, which could be attributed to the differences in pandemic time course and caseloads. Students who experienced a decline in sleep quality during the COVID-19 pandemic had higher dietary risk scores than students who did not experience a change in sleep quality (*p* = 0.001). Improved sleep quality was associated with less sitting time (*p* = 0.010). Addressing sleep issues among higher education students is a pressing concern, especially during stressful events. These results support the importance of making education and behavior-based sleep programming available for higher education students in order to benefit students’ overall health.

## 1. Introduction

Young adulthood is a time when many health behaviors solidify, making this developmental period a prime opportunity for public health practitioners to shape lifelong healthy habits [1]. While higher education student experiences vary across the globe, one important change from adolescence that many students face is increased independence in structuring one’s time [2,3,4]. In addition to academics, time is also spent on a variety of factors including socializing; working, volunteering, or extracurricular activities; and health behaviors like sleeping, eating and physical activity. Struggling with time management demands contributes to many undergraduate and graduate students reporting elevated levels of perceived stress [5,6]. These high levels of perceived stress are a global phenomenon; for example, prior to the pandemic more than three-quarters of Malaysian students reported experiencing moderate stress [7] while nearly 40% of USA students reported high levels of perceived stress [8]. Stress plays an important role in health behaviors, like insufficient sleep [6,9,10] and increased alcohol consumption [11,12,13], which can lead to negative health outcomes such as cardiovascular disease [14,15], diabetes [16,17] and cancers [18,19,20,21]. An improved understanding of the health behaviors of young adults, especially under conditions of heightened stress, is needed to assist public health practitioners in designing programming to promote the development of lifelong healthy habits.

While the prevalence of higher education students reporting elevated stress is high, the current COVID-19 pandemic has further increased stress for many students [22,23,24]. Previous studies demonstrated that large-scale disasters, including pandemics, led to profound health behavior changes [25,26], including unhealthy eating behaviors, poor sleep and lower physical activity levels [27,28,29,30,31,32,33]. Other studies noted increased mental health distress during disasters [34,35,36]. Because the student population is at increased risk for elevated stress and mental health concerns [37,38,39,40], and because many students experienced disruption of their established daily schedules, the current study sought to characterize higher education student health behaviors in multiple countries and to examine how these behaviors were affected by the COVID-19 pandemic.

During the COVID-19 pandemic, popular press and scholarly work reported that sleep duration and quality suffered [41,42,43]. The inability to obtain sufficient, good quality sleep has been demonstrated to be an independent risk factor for a variety of diseases and conditions, including obesity [44], type 2 diabetes [45,46], hypertension [45], alcohol misuse [47] and depression [48]. Stressful events, like a pandemic, can interfere with sleep [49]. Thus, the present study also explored whether changes in sleep quality and duration during the COVID-19 pandemic were related to health behaviors. It was hypothesized that a decline in sleep quality and a reduction in sleep duration would be associated with increased dietary risk scores and alcohol misuse, decreased physical activity levels and increased sitting time, while improved sleep quality and increased duration would be associated with the opposite.

## 2. Results

### 2.1. Demographic and Anthropometric Information

A total of 2663 students studying in China, Ireland, Malaysia, South Korea, Taiwan, the Netherlands and the USA initiated the survey, and a total of 2254 students completed it and were included in the analyses (Table 1). The majority of countries surveyed had more female participants except South Korea and Taiwan, and most of the students were undergraduate students except for South Korea. Students from all countries, besides the Netherlands, were predominantly domestic students, meaning that they attended a higher education institution in their country of citizenship. Students from the USA reported a higher body mass index (BMI) compared to all other countries. Overall, demographic information among the countries was fairly consistent, but BMI (based on self-reported height and weight) differed.

### 2.2. Differences in Health Behaviors by Country

Sleep quality and average, weekday and weekend sleep duration were largely consistent across countries. However, the highest and lowest absolute values often differed from each other (Table 2). Sleep quality was measured using the Pittsburgh Sleep Quality Index (PSQI) where a higher score indicates poorer sleep quality [50]. All countries reported PSQI scores greater than 5, which indicates poor sleep quality. The following sleep measures were significantly different between the highest and lowest absolute values reported, but these values did not differ between most other countries: PSQI score (USA vs. Taiwan, *p* < 0.001), average sleep duration (The Netherlands vs. Taiwan, *p* < 0.001) and weekday sleep duration (The Netherlands vs. Korea, *p* = 0.032). Additionally, the following proportions were significantly different between the highest and lowest absolute proportions, but these proportions did not differ between most other countries: the proportion of students classified as poor sleepers (USA vs. China, *p* < 0.05) and the proportion of students who did not meet the minimum recommended sleep duration of 7 h per day [51] (Korea vs. The Netherlands, *p* < 0.05). To summarize, sleep quality was poor for students from all countries and, in most countries, more than 25% of students were not meeting sleep duration guidelines.

Overall dietary risk and alcohol misuse scores were largely consistent by country, but the highest and the lowest absolute values were different from each other (Table 3). Students from the USA reported the highest absolute dietary risk scores, which differed from China where students reported the absolute lowest risk scores (*p* < 0.001). However, the two scores were not different from other countries. The following alcohol misuse measures were significantly different between the highest and lowest absolute values reported, but these values did not differ between most other countries: male students’ alcohol misuse scores (Ireland vs. Malaysia, *p* < 0.001) and the proportions of male students classified as alcohol misusers (Ireland vs. Malaysia, *p* < 0.05). Female students in China and Malaysia (*p* < 0.05 for both) reported a lower alcohol misuse score compared to all other countries, and the proportion of female students who were classified as misusers was lower in China and Malaysia (*p* < 0.05 for both) compared to all other countries.

Physical activity level, measured by self-reported total metabolic equivalents (METs) minutes per week, and by sitting time, displayed considerable consistency across countries; however, the highest and the lowest values differed from each other (Table 4). While Irish students were the most active, and Malaysian students the least (*p* < 0.001), activity levels reported by these two countries were not different from most other countries. In addition, students from Malaysia reported a higher amount of sitting time compared to most other countries.

### 2.3. Differences in COVID-19 Pandemic-Influenced Health Behaviors by Country

The influence of the COVID-19 pandemic on health behaviors was similar across countries with few differences observed (Table 5). The absolute proportion of students who indicated they were eating less healthfully during the pandemic compared to before was lowest in Taiwan compared to all other countries (*p* < 0.05), whereas the highest value, observed among USA students, did not differ among the remaining countries. The following behaviors were significantly different between the highest and lowest absolute values reported, but these values did not differ between most other countries: the proportion of students drinking more alcohol (USA vs. Malaysia, *p* < 0.05), sleeping worse and sleeping less (Ireland vs. Taiwan, *p* < 0.05) and exercising less (China vs. Ireland, *p* < 0.05). In addition, the proportion of students reporting decreased exercise intensity did not differ by country. Generally, in terms of health behaviors, students in European countries and the USA were more negatively affected by the COVID-19 pandemic during the study period compared to students in Asian countries.

### 2.4. Examination of Relationships between Sleep Quality and Duration Changes and Health Behaviors

PSQI scores and average sleep duration were compared between students who reported sleeping worse, sleeping better and no change in sleep quality during the COVID-19 pandemic compared to before. Students who reported sleeping worse during the pandemic experienced poorer sleep quality, as evidenced by higher PSQI scores, compared to students who reported that sleep quality did not change or who reported sleeping better (Table 6). Average sleep duration was different between the three groups, with students who reported sleeping better reporting the longest sleep duration while students who reported sleeping worse reported the shortest.

Sleep quality differed between students who reported sleeping less, sleeping more and no change in sleep duration during the COVID-19 pandemic compared to before. Students who reported sleeping less during the pandemic reported the highest PSQI scores (worse sleep quality), while students who reported better or no change in sleep duration reported lower PSQI scores that did not differ between each other (Table 6). As expected, average sleep duration differed between the three groups; those who reported sleeping less reported the shortest duration, those who reported sleeping more reported the longest duration, and those reporting no change reported an intermediate duration. Additionally, those who reported sleeping less had the highest PSQI scores compared to those who slept more and did not change in sleep duration.

Students who experienced reduced sleep quality during the pandemic had a higher dietary risk score compared to students who reported improved or no change in sleep quality (Table 7). Students who slept less and slept more had a higher dietary risk score compared to students who reported no change in sleep duration. To summarize, reduced sleep quality was associated with higher dietary risk scores, but change in sleep duration was not a reliable predictor of dietary risk.

Compared to students who reported no change in sleep quality, students who slept better and slept worse did not differ in alcohol misuse scores, and students who slept less and slept more both had higher alcohol misuse scores (Table 7). Therefore, change in sleep quality was not associated with alcohol misuse scores, and change in sleep duration was not a reliable predictor of alcohol misuse scores.

In terms of physical activity levels and sitting time, students who reported improved sleep quality reported less sitting time but no difference in physical activity levels compared to students who reported no change in sleep quality. There was no difference in physical activity levels and sitting time for students who reported reduced sleep quality when compared to students who reported no change (Table 7). Additionally, students who slept more reported lower physical activity levels and more sitting time, while there was no difference in physical activity levels and sitting time for students who slept less when compared to students who reported no change in sleep duration. To summarize, sleeping better during the COVID-19 pandemic was associated with higher physical activity levels and less sitting time, while sleeping more was associated with lower physical activity levels and more sitting time.

## 3. Discussion

Health behaviors of students across the seven countries were largely similar, although a few differences were noted. The negative effects of the COVID-19 pandemic on health behaviors disproportionately affected students in European countries and the USA compared to students in Asian countries. This likely resulted from the timeframe of the study (see discussion below). As hypothesized, students who indicated poorer sleep quality during the pandemic had higher dietary risk scores and engaged in less physical activity, while improved sleep quality was associated with higher physical activity levels and less sitting time. Contrary to our hypothesis, sleeping more during the pandemic was associated with lower physical activity levels and greater sitting time. These results suggest that working to improve the sleep quality of higher education students could be an important target for improving overall health by supporting healthier behaviors. This conclusion needs further testing due to the cross-sectional nature of the present study.

### 3.1. Differences in the Overall Influence of the COVID-19 Pandemic by Country

Students from European countries and the USA were generally more negatively affected by the COVID-19 pandemic compared to students in Asian countries, and students from Taiwan seemed to be the least negatively affected in terms of diet, alcohol consumption and sleep. The study took place between April and May 2020, during which COVID-19 cases were exponentially growing in the USA and in some European countries while the growth of cases had started to decline or plateau in China, Malaysia, South Korea and Taiwan [52]. Additionally, popular media reported that Taiwan had experienced just 690 novel coronavirus cases and seven deaths as of early December 2020, while the USA reported 14 million cases and more than 283,000 deaths [53]. As the case numbers grew in the USA and European countries and more deaths were being reported, students likely experienced increased stress. This supposition is supported by several studies that reported increased mental health concerns among higher education students in China during the Spring of 2020, when the peak number of COVID-19 cases in China emerged [54,55,56,57,58] and worsened mental health among higher education students and young adults in the USA reported both by popular media [59] and the Centers for Disease Control and Prevention [60]. Additionally, during the data collection period, the European countries and the USA enacted mandatory quarantine, which could also contribute to increased stress [61], while some Asian countries had lifted the lockdown order. These differences in pandemic-related stress could contribute to some of the differences observed between the countries surveyed.

### 3.2. Health Behaviors among Students in Higher Education during the COVID-19 Pandemic

Both insufficient sleep and poor sleep quality are health concerns among higher education students [62,63]. However, the present study observed that poor sleep quality is a more immediate problem for this population. The average PSQI score for all countries was greater than 5, which is consistent with sleep problems [64] and consistent with previous reports regarding high prevalence of poor sleep quality among higher education students [12,65,66,67]. Sleep duration was less of a concern than sleep quality for most countries except South Korea and Taiwan, where more than 40% of students failed to meet the minimal recommended sleep guidelines of 7 h per night. Unlike other reports [52,57,58], more students in the present study met the recommended minimum sleep duration guidelines of 7 h. For example, one meta-analysis of Chinese students’ sleeping patterns reported 43.9% of students failing to sleep at least 7 h per day [67], but the prevalence of insufficient sleep among our Chinese cohort was only 14.4%. Another study, where the majority of students were from the Netherlands, revealed that nearly 25% of students slept less than 7 h [68]. This contrasts with the present findings, where 12.3% of students in the Netherlands did not meet minimum recommendations. Further, a study of higher education students in Malaysia revealed that nearly 60% of students reported sleeping less than 7 h per day compared to the present study’s findings of 34.1% [62]. The differences in sleeping duration results observed in the present study compared to previously published work could be attributed to the large proportion of students (37.0–55.9% across the different countries) in the present study who reported sleeping more during the COVID-19 pandemic compared to before. While getting an adequate amount of sleep is important, improving sleep quality among students in higher education appears to be a more pressing concern, especially during a stressful event like the COVID-19 pandemic.

Dietary risk scores of students were largely consistent across all countries with few differences observed. These findings are consistent with the current literature [69,70,71,72,73,74,75,76]. Students from the USA reported the highest absolute dietary risk score, which is consistent with the higher prevalence of overweight and obesity among American students, and higher BMI compared to students in the other countries studied [69,70,71,72,73,74,75,76]. Even though the prevalence of overweight and obesity was higher among USA students, previous work reported that the majority of students from the USA and other countries did not meet the daily recommended servings of fruit and vegetable intake [77,78,79,80,81]. Additionally, approximately one in three students in the present study reported eating less healthfully during the COVID-19 pandemic compared to before, and almost half of the USA students reported eating less healthfully during the COVID-19 pandemic. These findings suggest poor dietary behaviors among students in higher education are global issues, and the COVID-19 pandemic appears to have worsened the problem for a substantial proportion of students.

While a lower proportion of students were classified as alcohol misusers compared to other recent reports [71,82,83,84,85], the findings still warrant concern. For example, a recent report observed two-thirds of Irish students misused alcohol (classified as an AUDIT-C score of greater than six among male students and greater than five among female students by the study’s authors) [71], while the present study reported half of both male and female Irish students were alcohol misusers based on AUDIT-C cut-off scores of greater than four for male and three for female according to the National Institute on Drug Abuse [86,87,88]. Given these lower cut-offs, there is considerable discrepancy between the current study’s findings and those of the Davoren group [71]. Further, while the current results classified 48.1% of male and 42.9% of female South Korean students as alcohol misusers, others reported 65% of South Korean students were problem drinkers [85]. It is possible that the pandemic played a role in these differences, as one of the primary reasons for drinking among students involves socializing [89,90]. The lower prevalence of alcohol misuse could be due, in part, to social distancing rules related to the pandemic that reduced students’ ability to socialize. Additionally, it could also due to changes in living situations, as COVID-19 caused 36% of college students to move, which could contribute to reduced opportunities to socialize [91]. Despite the reduced prevalence of problem drinking in this sample, alcohol misuse continues to be a grave concern in many countries.

The absolute total METs/week of students in Ireland met the recommended 3000 to 4000 METs per week for disease prevention and health improvement [92], while students in other countries did not. These results were lower than what was previously reported in European countries and the USA but similar to what was reported in Asian countries; for example, students in the USA reported an average physical activity of 6000 METs/week [93], and students in several European countries reported approximately 3500 to 4600 METs/week [94], while students in China reported just over 1000 METs/week [95]. During the COVID-19 pandemic, approximately half of students in the present study reported lower physical activity levels, which could explain the discrepancy between physical activity levels reported in the current study and previous studies. Overall, the COVID-19 pandemic appeared to negatively influence many students’ physical activity levels.

### 3.3. Health Behavior Analysis

The present study observed that students reporting decreased sleep quality during the pandemic reported higher dietary risk scores, while students reporting improved sleep quality reported less siting time. The results are consistent with previous studies reporting that higher education students with poor sleep quality consumed less fruit and dairy servings, and poor sleep quality was associated with higher energy intake and poorer diet quality among adult women including young adults [96,97]. Poor sleep, including both insufficient and poor quality sleep, may also contribute to low physical activity levels [98,99,100,101] and is associated with sedentary behaviors [102,103]. Therefore, dietary habits and physical activity levels of students are likely to be improved when students’ sleep quality improves.

While both sleep duration and quality are important for health [97,98,99,100,101,103,104], the current study observed that poor sleep quality appeared to be a more critical risk factor to health than sleep duration. Most literature suggests that sufficient sleep is associated with a higher quality diet and higher physical activity levels [98,99,100,101,104], but the current study found that sleep duration change was not consistently related to dietary risk, and sleeping more was associated with lower physical activity levels and more sitting time. These discrepancies might be explained by the high PSQI scores (>6) reported by all three sleep duration change groups, as higher PSQI scores are associated with unhealthy dietary behaviors [97,103], less physical activity and more sedentary behaviors [99,105]. Additionally, the present study observed that nearly 45% of students reported sleeping more during COVID-19 and 32% of students reported sleeping worse. It is possible that some students compensated for poor sleep quality with increased duration, as previous work reported that longer sleep duration was related to poor subjective sleep quality [106]. Therefore, in the present study sample, sleep quality change, rather than sleep duration change, appears to be more closely associated with dietary behaviors and sitting time.

Alcohol misuse is common among higher education students [71,83,107], and previous work indicated college students who reported poor sleep quality consumed alcohol more frequently and excessively and experienced more negative consequences [108,109,110,111,112]. Additionally, insufficient sleep impairs cognitive functioning that serves as a protective mechanism against alcohol misuse [113,114,115,116]. The present study, however, found that changes in sleep quality and duration did not predict higher or lower alcohol misuse scores, which could be explained by the high PSQI scores of all groups compared, as PSQI scores and sleep duration were negatively correlated in this study. Given that all groups compared for changes in sleep duration and quality reported a PSQI score above 5, differences in alcohol misuse might not be detectable.

### 3.4. Public Health Message

While sleeping adequately is important for health, enhancing sleep quality is also critical; yet, improving higher education students’ sleep quality has received little attention [117,118]. Higher education students in all countries studied suffered from poor sleep quality, which has been associated with lower grades, impaired learning ability, worse mood, greater stress, more risk-taking behaviors and poorer overall health [10,108,119,120,121]. One study of over 55,000 students reported that stress, binge drinking and drug use had similar, or even smaller, associations with academic success compared to sleep disturbance; yet these risk factors received far more attention by university administrators compared to sleep problems [121]. Improved sleep quality of students is associated with not only better health but also better academic performance, improved graduation rates and improved future earnings [117]. Further, one study predicted that the economic gain of improving students’ sleep exceeds the cost of implementing sleep education programs [117]. Thus, improving sleep among students can likely serve as an effective and efficient way to improve students’ future health and economic success.

A variety of strategies can be adopted by universities to improve students’ sleep, including sleep hygiene education [122,123], cognitive behavioral therapy for insomnia (CBTi), relaxation, mindfulness and hypnotherapy [124]. CBTi is an intervention that involves cognitive, behavioral and educational interventions [125]. CBTi focuses on helping individuals suffering from insomnia to identify and modify thoughts, feelings and behaviors that contribute to sleep problems. Two systematic reviews have concluded that CBTi-based sleep interventions delivered the largest improvements in a multitude of sleep measures among college students compared to other interventions [124] and that online delivery of CBTi is effective [126]. Given the ongoing global pandemic and social distancing requirements in many countries, an online CBTi-based sleep education program could be a feasible way to improve students’ sleep especially when more than 30% of students reported sleeping worse during the pandemic. Ensuring sufficient, good quality sleep not only protects against a variety of chronic diseases [44,45,46] but also against more immediate health concerns like poor diet quality [97], mental health concerns [48] and alcohol misuse [47]. Therefore, university administrators should consider implementing effective strategies, such as CBTi, among university students to improve sleep quality and promote the overall health of students.

### 3.5. Strengths and Limitations

There are several strengths to this study. First, the study involved a large sample of students from seven different countries on three continents, which increases the generalizability of the results. Second, the study utilized validated surveys for assessing health behaviors of higher education students. Third, the health behavior differences by country were controlled for in the linear regression models. Fourth, the study included both undergraduate and graduate students, which increased the generalizability of the results to a larger student body.

There are limitations to the study. First, the cross-sectional nature of the study precludes conclusions about the causal relationships between sleep changes and health behaviors changes. Second, the COVID-19 questions included in the survey were not validated; however, validated questionnaires were not available for addressing the specific questions in the study due to the emerging nature of the pandemic. Third, countries included in the study were under different quarantine rules and stages of the pandemic during the data collection period, and these differences could contribute to disparities in and differences in health behaviors. Finally, students who took the study survey were required to have English proficiency, which might have resulted in not all students who wished to participate being able to do so.

## 4. Materials and Methods

### 4.1. Study Design

Higher education students, including both undergraduate and graduate students, who were at least 18 years old and were enrolled in universities in China, Ireland, Malaysia, Taiwan, South Korea, the Netherlands and the USA were recruited into this cross-sectional study. Countries included in the present study were based on availability. Survey data was collected in April and May 2020, when most states in the USA had adopted shelter in place orders, and most areas in Ireland, Malaysia, and the Netherlands had also enacted shelter in place orders due to the COVID-19 pandemic. During this time, China, Taiwan, and South Korea had recently revoked their shelter in place orders.

The study was approved by the Michigan State University Human Research Protection Program (East Lansing, MI, USA) STUDY00004285, 7 April 2020; International Medical University Joint Committee on Research and Ethics (Kuala Lumpur, Malaysia), 481/2020, 14 May 2020; Faculty of Governance and Global Affairs Ethics Committee (The Hague, South Holland, The Netherlands), 2020-009-LUC-Cho, 25 May 2020; Indiana University Institutional Review Board for the Protection of Human Subjects (Indiana, PA, USA), IRB Log 20-101, 15 May 2020; Institute Research Ethics Committee, Institute of Technology, Sligo (Sligo, Ireland), ref 2020015, 5 May 2020; University of Taipei Institutional Review Board (Taipei, Taiwan), IRB-2020-045, 16 June 2020; and Bowling Green State University Office of Research Compliance (Bowling Green, OH, USA) 15,997,53 (US students), 29 April 2020; 1,599,753 (Chinese students), 22 May 2020; 1,599,753 (Korean students), 11 May 2020. Consent was obtained from all participants prior to the start of the study.

### 4.2. Demographics

Information regarding age, gender, undergraduate vs. graduate status including year classification, domestic versus international status, weight and height was collected.

### 4.3. Assessment of Dietary Risk and Alcohol Misuse

Diet was evaluated using the Starting the Conversation (STC) questionnaire. The STC is an eight-item simplified food frequency questionnaire (FFQ), which was used to determine participants’ dietary risk score based on common dietary patterns [127]. Examples of questions include: How many times a week did you eat fast food meals or snacks? How many regular sodas or glasses of sweet tea did you drink each day? The STC provides a global score of dietary risk, with higher scores indicating greater risk. Food terms were adjusted to account for cultural differences among countries. For example, the term snack chips was changed to crisps in the Ireland survey.

Alcohol misuse score was determined using the Alcohol Use Disorders Identification Test Alcohol Consumption questionnaire (AUDIT-C), which is a validated screener for problem drinking over the past year [86]. A standard drink equivalents reference chart was included in the survey to help participants quantify drink servings [128]. A score of 4 and higher for men and 3 and higher for women suggests misuse [129]. The alcohol assessment questions were only shown to students who were of legal drinking age in their respective countries.

### 4.4. Assessment of Sleep Quality and Duration

Assessment of sleep quality and duration over the past month was conducted using the Pittsburgh Sleep Quality Index (PSQI), which has been validated [64,130,131,132]. In addition to the PSQI, weekday and weekend sleep duration were also quantified in order to distinguish sleeping duration differences between weekdays and weekends.

### 4.5. Assessment of Physical Activity and Sitting Time

Physical activity levels and sitting over the past week were measured using the International Physical Activity Questionnaire (IPAQ) [133]. The total physical activity levels were calculated and reported using metabolic equivalents (METs) minutes per week. The total METs minutes per week were calculated using METs of vigorous, moderate, or walking activity multiplied by the corresponding activity duration. Therefore, METs minutes per week reflects both physical activity intensity and duration. Total METs minutes per week was selected to reflect the overall physical activity levels of participants as the measure captures both physical activity intensity and duration. As such, physical activity level is used to refer to METs minutes per week. Sitting times were reported as minutes per day.

### 4.6. Assessment of the Influence of the COVID-19 Pandemic on the Factors Described Above

At the end of each survey section, a question about how the COVID-19 pandemic had affected participants’ diet, alcohol consumption, sleep quality, sleep duration and physical activity frequency and intensity was asked. Examples of questions included: Have you made changes to your diet during the COVID-19 quarantine compared to before the quarantine? Possible answers included: healthier than before, less healthy than before or my diet did not change. Has the intensity of your exercising changed during the COVID-19 quarantine compared to before the quarantine? Possible answers included: more intense, less intense, or my exercise intensity did not change.

### 4.7. Statistical Analysis

Outlier screening was conducted for each variable, and outliers defined as greater or less than mean values ± 3 standard deviations, were excluded from data analysis. After outlier exclusion, all examined variables were approximately normally distributed based on normal probability plot, kurtosis and skewness. Adjusted totals were calculated by averaging variable means of each country to account for differences in country sample size [134]. Differences between countries for age, BMI, sleep quality and duration, dietary risk, alcohol misuse risk, physical activity and sitting time were assessed using one-way ANOVA and Bonferroni post hoc tests. Statistical significance for these tests was determined by *p* < 0.05. Categorical variables of gender, undergraduate vs. graduate status, domestic versus international status and health behaviors influenced by the COVID-19 pandemic were compared between countries using a chi-square test of homogeneity followed by z-test comparisons of country proportions. Bonferroni adjusted *p*-values were used to determine significance. Specific *p* values for proportion comparisons were not given by SPSS. Therefore, the *p* value cut-offs were calculated based on Bonferroni adjustment rules of alpha divided by number of comparisons [135,136]. Linear regression with dummy coding of categorical independent variables was conducted to examine whether sleep quality and duration changes were related to dietary risk scores, alcohol misuse scores, physical activity levels (duration and frequency reflected by total METs per week) and sitting time. The differences in PSQI scores and sleep duration were compared between the sleep quality change groups and between the sleep duration change groups using one-way ANOVA. Normal P-P plot and scatter plots of regression standardized residuals were produced, and the plots showed that assumptions of normality and homoscedasticity were met. Linear relationships between independent variables and the outcome variable were observed. Four linear regression models adjusting for age, gender, BMI and countries were built using the Enter method. All analyses were completed using IBM SPSS version 26 (IBM Corporation, Armonk, NY, USA).

## 5. Conclusions

The present study identified numerous similarities and only a few differences in health behavior risks among higher education students living in seven different countries. Sleeping worse was associated with higher dietary risk scores while sleeping better was associated with less sitting time. Collectively, these data demonstrate that poor sleep quality is a pressing concern among higher education students around the world, especially during the COVID-19 global pandemic. Based on previous work, online CBTi-based sleep education programs appear to be a socially distanced and relatively inexpensive way to improve students’ sleep outcomes and overall health.

## Figures and Tables

**Table 1:** Demographic and anthropometric information of higher education students from 7 countries.

	China	Ireland	Malaysia	South Korea	Taiwan	The Netherlands	USA	Total	Adjusted Total
*N*	111	192	91	89	377	114	1280	2254	-
Female (%)	67.6 ^a^	71.9 ^a^	79.1 ^a^	39.3 ^b^	41.1 ^b^	80.7 ^a^	73.0 ^a^	66.6 ^†^	74.5
Male (%)	32.4 ^a^	27.6 ^a^	20.9 ^a^	60.7 ^b^	53.6 ^b^	19.3 ^a^	24.1 ^a^	30.8 ^†^	33.7
Age (year) (Mean ± SD)	20.4 ± 2.5 ^c^	24.7 ± 7.6 ^a^	22.2 ± 4.1 ^b,c^	25.5 ± 4.6 ^a^	20.8 ± 3.1 ^c^	20.1 ± 1.3 ^c^	22.9 ± 5.9 ^b^	22.5 ± 5.5	22.4
BMI (kg/m^2^) (Mean ± SD)	20.2 ± 2.9 ^d^	24.1 ± 6.3 ^b^	22.2 ± 4.6 ^b,c,d^	22.7 ± 3.4 ^b,c^	22.5 ± 3.2 ^c^	22.0 ± 3.6 ^c,d^	26.0 ± 6.4 ^a^	24.4 ± 5.6	22.8
Undergraduate (%)	84.7 ^a,b,c^	80.2 ^c^	96.7 ^b,d^	46.1 ^e^	95.5 ^d^	100.0 ^d^	74.3 ^a,c^	79.9	88.6
Domestic student (%)	95.5 ^a,b^	93.2 ^a,b^	81.3 ^c^	94.4 ^a,b,c^	95.5 ^b^	40.4 ^d^	87.0 ^a,c^	87.0	91.2

Means with different superscripts are significantly different, *p* < 0.05. Chi-square test of homogeneity was used for comparisons of gender, undergraduate vs. graduate status, and domestic vs. international status. One-way ANOVA was used for comparisons of age and BMI. Domestic student was defined as a student attending a higher education institution in their country of citizenship. ^†^ 2.6% of students self-identified as other, which included transgender, genderqueer, other, and choose not to disclose. Missing values: gender *n* = 0; age *n* = 2; BMI *n* = 16; undergraduate vs. graduate status *n* = 0; domestic vs. international status *n* = 0.

**Table 2:** Country comparisons of sleep quality and duration.

	China	Ireland	Malaysia	South Korea	Taiwan	The Netherlands	USA	Total *	Adjusted Total
*N*	111	190	91	89	375	114	1272	2242	-
PSQI (Mean ± SD)	5.5 ± 2.6 ^b,c^	7.4 ± 3.6 ^a^	5.9 ± 3.1 ^b,c^	6.4 ± 3.1 ^a,b,c^	5.5 ± 3.1 ^c^	6.8 ± 3.4 ^a,b^	7.4 ± 3.6 ^a^	6.8 ± 3.5	6.4
Average sleep (h/d) (Mean ± SD)	7.7 ± 0.8 ^a,b^	7.5 ± 1.2 ^a,b,c^	7.2 ± 1.2 ^b,c,d^	7.1 ± 1.1 ^c,d^	7.0 ± 1.1 ^d^	7.7 ± 1.0 ^a^	7.6 ± 1.3 ^a^	7.5 ± 1.2	7.5
Weekday sleep (h/d) (Mean ± SD)	7.5 ± 0.9 ^a,b^	7.3 ± 1.2 ^a,b^	7.0 ± 1.2 ^a,b^	6.7 ± 1.3 ^b^	7.0 ± 1.3 ^b^	8.2 ± 4.6 ^a^	7.6 ± 2.5 ^a,b^	7.5 ± 3.5	7.4
Weekend sleep (h/d) (Mean ± SD)	8.2 ± 1.1	8.0 ± 1.4	7.8 ± 1.5	8.3 ± 1.5	8.2 ± 3.4	8.2 ± 1.1	8.2 ± 1.6	8.1 ± 1.9	8.1
Poor sleep quality (%)	46.8 ^a,b,c,d^	64.7 ^c,d,e^	49.5 ^a,b,c,d^	60.7 ^b,d,e^	42.7 ^a^	55.3 ^a,b,c,d,e^	67.2 ^e^	60.3	55.3
Short sleep duration (%)	14.4 ^a^	26.6 ^a,b^	34.1 ^b,c^	46.1 ^c^	43.2 ^c^	12.3 ^a^	24.2 ^a,b^	27.8	28.7

Means with different superscripts are significantly different, *p* < 0.05. One-way ANOVA was used for comparisons of PSQI, average sleep hours, weekday and weekend sleep hours. Chi-square test of homogeneity was used for comparisons of percentages of students who reported poor sleep quality and short sleep duration. PSQI = Pittsburg Sleep Quality Index. PSQI scores range from 0 = best to 21 = worst sleep quality. PSQI scores > 5 were defined as poor sleep quality. Short sleep duration was defined as sleep duration <7 h/day (average of weekday and weekends). * Included all valid data for which PSQI scores could be computed. Missing values: PSQI *n* = 12; average sleep duration *n* = 21; weekday sleep duration *n* = 2; weekend sleep duration *n* = 2; poor sleep quality (%) *n* = 12; short sleep duration (%) *n* = 21.

**Table 3:** Country comparisons of dietary risk and alcohol consumption.

	China	Ireland	Malaysia	South Korea	Taiwan	The Netherlands	USA	Total *	Adjusted Total
*N*	111	192	91	89	-	114	1280	1877	-
Dietary risk (Mean ± SD)	6.6 ± 2.3 ^b^	6.9 ± 3.1 ^b^	7.1 ± 2.4 ^b^	7.4 ± 2.9 ^a,b^	-	7.0 ± 2.5 ^b^	8.2 ± 2.7 ^a^	7.9 ± 2.8	7.3
*n* (provided alcohol use data)	107	190	91	86	377	114	796	1761	-
Alcohol misuse score (Mean ± SD) (male ^†^)	2.3 ± 2.5 ^c^	4.4 ± 2.5 ^a^	1.2 ± 1.6 ^c^	4.2 ± 3.3 ^a,b^	4.0 ± 3.3 ^a,b^	4.4 ± 2.3 ^a,b^	3.3 ± 3.0 ^b^	3.6 ± 3.1	3.4
Alcohol misuse score (Mean ± SD) (female ^††^)	1.2 ± 1.7 ^a^	3.6 ± 2.1 ^b^	0.9 ± 1.4 ^a^	3.1 ± 2.8 ^b,c^	2.7 ± 2.4 ^c^	3.7 ± 2.3 ^b^	3.1 ± 2.4 ^b,c^	2.9 ± 2.4	2.6
Classified as misuser (%) (male ^†^)	25.0% ^a,b,c,d^	49.1% ^d^	5.3% ^c^	48.1% ^b,d^	42.6% ^b,d^	45.5% ^a,b,c,d^	23.7% ^a,c^	33.3%	34.2%
Classified as misuser (%) (female ^††^)	8.0% ^a^	50.7% ^b^	6.9% ^a^	42.9% ^b,c^	36.8% ^b^	53.3% ^b^	23.3% ^c^	28.0%	31.7%

Means with different superscripts are significantly different, *p* < 0.05. ^†^ For male students, *n* = 35 China, *n* = 53 Ireland, *n* = 19 Malaysia, *n* = 54 South Korea, *n* = 202 Taiwan, *n* = 22 the Netherlands, *n* = 211 the USA ^††^ For female students, *n* = 72 China, *n* = 136 Ireland, *n* = 72 Malaysia, *n* = 32 South Korea, *n* = 155 Taiwan, *n* = 92 the Netherlands, *n* = 561 the USA Dietary risk was defined by score on the Starting the Conversation food frequency questionnaire. 0 = best to 16 = worst dietary quality. Alcohol misuse was defined by score on the Alcohol Use Disorders Identification Test Consumption questionnaire. 0 = no alcohol use to 12 = highest alcohol use. Scores ≥ 3 in females and ≥4 in males were defined as misuse. * Missing values for dietary risk, *n* = 377. Dietary risk data from Taiwan were not collected due to technical difficulties. Missing values for alcohol misuse, *n* = 493, represented students who did not meet the legal drinking age of their country.

**Table 4:** Country comparisons of physical activity level and sitting time.

	China	Ireland	Malaysia	South Korea	Taiwan	The Netherlands	USA	Total *	Adjusted Total
*N*	105	177	88	87	-	109	1174	1742	-
Total METs (min/wk) (Mean ± SD)	1966.5 ± 2710.9 ^b,c^	3748.1 ± 3772.3 ^a^	1639.2 ± 2502.9 ^c^	2842.6 ± 3317.9 ^a,b,c^	-	2849.7 ± 2442.6 ^a,b,c^	2892.9 ± 3549.5 ^b^	2859.8 ± 3441.0	2656.5
Sitting time (min/d) (Mean ± SD)	285.7 ± 204.4 ^a^	389.7 ± 189.7 ^b^	492.8 ± 255.5 ^c^	368.5 ± 200.2 ^a,b^	-	427.1 ± 165.5 ^b,c^	423.8 ± 204.3 ^b^	413.0 ± 206.7	397.9

Means with different superscripts are significantly different, *p* < 0.05. One-way ANOVA was used for comparisons of total METs and sitting time. Physical activity level was defined by metabolic equivalents (METS) reported on the International Physical Activity Questionnaire. * Physical activity data from Taiwan were not collected due to technical difficulties.

**Table 5:** Percentage of students reporting undesirable changes in health behaviors during the COVID-19 pandemic compared to before the pandemic.

		China (%)	Ireland (%)	Malaysia (%)	South Korea (%)	Taiwan (%)	The Netherlands (%)	USA (%)	Total	Adjusted Total
*N*		111	192	91	89	377	114	1280	2254	-
Diet	Less healthy	22.5 ^a^	35.8 ^a,b^	36.3 ^a,b^	24.7 ^a^	6.1 ^c^	33.3 ^a,b^	45.6 ^b^	35.2	30.5
Alcohol consumption *	Drinking more	5.6 ^a,b,c^	16.3 ^c,d^	2.2 ^b^	5.8 ^a,b,c^	6.1 ^b^	17.5 ^a,b,d^	26.1 ^d^	16.8	8.9
Sleep quality	Sleeping worse	16.2 ^a,b^	41.6 ^c^	29.7 ^b,c^	19.1 ^b^	8.0 ^a^	48.2 ^c^	38.8 ^c^	32.1	21.7
Sleep duration	Sleeping less	12.6 ^a,b,c,d,e^	27.4 ^d,e^	22.0 ^c,e^	10.1 ^a,b,c^	8.2 ^b^	20.2 ^a,c,d,e^	18.5 ^a,c,d,e^	17.1	17.0
Exercise frequency *	Exercising less	56.8 ^a^	37.0 ^b^	50.5 ^a,b^	44.9 ^a,b^	-	50.0 ^a,b^	51.5 ^a^	49.9	48.5
Exercise intensity *	Less intense	49.5 ^a^	39.7 ^a^	42.9 ^a^	38.2 ^a^	-	43.0 ^a^	46.4 ^a^	45.2 ^a^	43.3

Means with different superscripts are significantly different, *p* < 0.05. Chi-square test of homogeneity was used for comparisons of all variables presented in the table. * *n* different from the *n* presented in the table. Total *n* for alcohol = 1761 as alcohol consumption questions were only shown to students who were of legal drinking age. Total *n* for exercise frequency and intensity = 1873. Exercise data from Taiwan were not collected due to technical difficulties.

**Table 6:** PSQI and average sleep duration of sleep quality and duration change groups.

Groups	*n* (%)	PSQI (Mean ± SD)	Average Sleep Duration (h/day) (Mean ± SD)
**Sleep quality change**			
Worse	719 (32.1)	9.2 ± 3.4 ^a^	7.3 ± 1.3 ^a^
Better	348 (15.5)	5.5 ± 2.9 ^b^	7.8 ± 1.1 ^b^
Did not change	1175 (52.4)	5.8 ± 2.9 ^b^	7.5 ± 1.1 ^c^
**Sleep duration change**			
Less	383 (17.1)	9.4 ± 3.8 ^a^	6.7 ± 1.2 ^a^
More	1002 (44.7)	6.6 ± 3.2 ^b^	8.0 ± 1.1 ^b^
Did not change	857 (38.2)	6.0 ± 3.2 ^c^	7.3 ± 1.0 ^c^

Means with different superscripts are significantly different, *p* < 0.05. PSQI = Pittsburgh Sleep Quality Index.

**Table 7:** Outcomes of linear regression models examining whether sleep changes were related to health behaviors.

Predictors	Dietary Risk B (*p*-Value)	Alcohol Misuse Scores B (*p*-Value)	Physical Activity (METs/wk) B (*p*-Value)	Sitting Time (min/d) B (*p*-Value)
(Constant)	6.756 (<0.001)	2.005 (<0.001)	2517.932 (<0.001)	368.664 (<0.001)
Sleep quality change				
Worse	0.486 (0.001)	0.310 (0.051)	−272.907 (0.123)	19.909 (0.082)
Better	−0.323 (0.081)	10.162 (0.413)	18.737 (0.934)	−36.064 (0.010)
Did not change (reference)	-	-	-	-
Sleep duration change				
Less	0.409 (0.040)	0.473 (0.019)	−128.772 (0.582)	4.436 (0.770)
More	0.408 (0.008)	0.459 (0.004)	−568.192 (0.002)	22.841 (0.050 *)
Did not change (reference)	-	-	-	-

*n* = 1877 for dietary risk, *n* = 1761 for alcohol misuse scores, *n* = 1736 for physical activity, and *n* = 1872 for sitting time. One model was built for each dependent variable, and the models controlled for age, BMI, gender, and countries. * The exact *p* value is 0.049880. Dietary risk was defined by score on the Starting the Conversation, a simplified food frequency questionnaire. 0 = best to 16 = worst dietary quality. Alcohol misuse was defined by score on the Alcohol Use Disorders Identification Test Consumption questionnaire. 0 = no alcohol use to 12 = highest alcohol use. Scores ≥ 3 in females and ≥4 in males were defined as misuse. Physical activity level and sitting time were reported on the International Physical Activity Questionnaire. METs = metabolic equivalents.

## Data Availability

The data presented in this study are available on request from the corresponding author. The data are not publicly available due to ongoing analyses.
